# Using data from ‘visible’ populations to estimate the size and importance of ‘hidden’ populations in an epidemic: A modelling technique

**DOI:** 10.1016/j.idm.2020.09.007

**Published:** 2020-09-30

**Authors:** Anna M. Foss, Holly J. Prudden, Kate M. Mitchell, Michael Pickles, Reynold Washington, Anna E. Phillips, Michel Alary, Marie-Claude Boily, Stephen Moses, Charlotte H. Watts, Peter T. Vickerman

**Affiliations:** a Department of Global Health and Development and Centre for Mathematical Modelling of Infectious Diseases, London School of Hygiene and Tropical Medicine, 15-17 Tavistock Place, London, WC1H 9SH, UK; b Department of Infectious Disease Epidemiology, School of Public Health, Imperial College London, Medical School Building, St Mary’s Campus, Norfolk Place, London, W2 1PG, UK; c St John’s Research Institute, 100 Feet Road, John Nagar, Koramangala, Bangalore, 560 034, Karnataka, India; d Department of Community Health Sciences, Max Rady College of Medicine, Rady Faculty of Health Sciences, University of Manitoba, S113-750 Bannatyne Avenue, Winnipeg, Manitoba, R3E 0W3, Canada; e Centre de recherche du CHU de Québec – Université Laval, 1050 Chemin Ste-Foy, Québec (Qc), G1S 4L8, Canada; f Département de médecine sociale et préventive, Faculté de médecine, Université Laval, 1050, avenue de la Médecine, Québec (Qc), G1V 0A6, Canada

**Keywords:** HIV, Infectious diseases, Mathematical modelling, Men who have sex with men, Transgender women, India, MSM/TGW, cisgender men or transgender women, who have sex with cisgender men or transgender women, IBBA, integrated biological and behavioural assessment survey, ART, antiretroviral therapy, SBS, special behavioural survey, FSW, female sex worker, PB, *panthis* and bisexuals

## Abstract

We used reported behavioural data from cisgender men who have sex with men and transgender women (MSM/TGW) in Bangalore, mainly collected from ‘hot-spot’ locations that attract MSM/TGW, to illustrate a technique to deal with potential issues with the representativeness of this sample.

A deterministic dynamic model of HIV transmission was developed, incorporating three subgroups of MSM/TGW, grouped according to their reported predominant sexual role (insertive, receptive or versatile). Using mathematical modelling and data triangulation for ‘balancing’ numbers of partners and role preferences, we compared three different approaches to determine if our technique could be useful for inferring characteristics of a more ‘hidden’ insertive MSM subpopulation, and explored their potential importance for the HIV epidemic.

Projections for 2009 across all three approaches suggest that HIV prevalence among insertive MSM was likely to be less than half that recorded in the surveys (4.5–6.5% versus 13.1%), but that the relative size of this subgroup was over four times larger (61–69% of all MSM/TGW versus 15%). We infer that the insertive MSM accounted for 10–20% of all prevalent HIV infections among urban males aged 15–49.

Mathematical modelling can be used with data on ‘visible’ MSM/TGW to provide insights into the characteristics of ‘hidden’ MSM. A greater understanding of the sexual behaviour of all MSM/TGW is important for effective HIV programming. More broadly, a hidden subgroup with a lower infectious disease prevalence than more visible subgroups, has the potential to contain more infections, if the hidden subgroup is considerably larger in size.

## 1 Introduction

HIV prevalence has declined in southern India in recent years (National AIDS Control Organisation, 2017b), thought to be attributed in part to reductions in heterosexual transmission during commercial sex (Arora, Kumar, Bhattacharya, Nagelkerke, & Jha, 2008). However HIV prevalence remains high among gay, bisexual and other men who have sex with men (MSM) and people who inject drugs (Solomon et al., 2015, 2019). In fact, in three northern cities of India there is the suggestion of emerging epidemics among MSM as HIV incidence is high in that population, despite low or moderate HIV prevalence in the general population (Solomon et al., 2015).

In India, concepts of gender and sexual role have historically defined different subgroups (Asthana & Oostvogels, 2001), with identity linked to whether they predominantly take the receptive (passive) or insertive (active) role in anal sex (Phillips et al., 2008). We use the acronym MSM to refer to cisgender men who have sex with other cisgender men or transgender women, and the acronym TGW to refer to transgender women who have sex with MSM. Examples of these MSM/TGW subgroups include the following:

• *Hijras* are transgender women, predominantly take the receptive role during anal sex and have exclusively male partners.
• *Kothis* are cisgender men and predominantly take the receptive role during anal sex.
• Double-deckers are cisgender men who are more likely to have sex with other men than women (Phillips, Bradley, Lowndes, Anthony, & Alary, 2009) and are versatile (have no preference between insertive and receptive roles) in anal sex.
• Bisexuals are cisgender men who are predominantly insertive and are thought to prefer sex with men but also engage in sex with women (Phillips et al., 2009).
• *Panthis* are cisgender men who engage in sexual activity with other men but do not identify as MSM (although are included within our use of the term MSM). They are thought, more typically, to take the insertive role and to have women as their main sexual partners.

As part of *Avahan*, the India AIDS Initiative of the Bill & Melinda Gates Foundation, two rounds of integrated biological and behavioural assessment (IBBA) surveys were conducted among MSM/TGW in the city of Bangalore, within Karnataka state in southern India, the first in 2006 and the second in 2009 (Bill & Melinda Gates Foundation, 2008; Brahmam et al., 2008; Karnataka Health Promotion Trust, 2013; Saidel et al., 2008). The majority of the data were collected from MSM/TGW recruited at city ‘hot-spot’ cruising sites, with over half of those surveyed reporting sex involving payment (Brahmam et al., 2008; Karnataka Health Promotion; Saidel et al., 2008; Karnataka Health Promotion Trust, 2013). Of those surveyed in 2009, 85% reported their identity as being either *hijras*, *kothis* or double deckers and 78% of the total sample reported their main sexual partner as being either a *panthi* or bisexual (Karnataka Health Promotion Trust, 2013). Consequently, *panthis* and bisexuals are likely to be significantly under-represented in the surveys.

A mapping and enumeration exercise were undertaken prior to the surveys which provided MSM population size estimates (Bill & Melinda Gates Foundation, 2008; Lowndes et al., 2012), but these were considered to be underestimates and predominantly identified *hijras* and *kothis*. It is, therefore, unclear how large the population of *panthis* and bisexuals may be, and how representative the *panthis* and bisexuals included in the surveys are of their wider population. Such information is important for understanding optimal HIV prevention strategies and whether these should focus predominantly on the ‘visible’ MSM/TGW (i.e. MSM/TGW captured in the surveys at hot spots), or expand outreach to also include the MSM who are currently more ‘hidden’ (i.e. *panthis* and bisexuals that may not have been adequately captured in the surveys). It is thought that a large proportion of MSM are likely to be ‘hidden’ from society, and do not participate in intervention services (Phillips et al., 2010).

The objectives of this paper are to investigate whether our mathematical modelling and data triangulation techniques can be used to determine if the epidemiological attributes of the *panthis* and bisexuals captured in the survey are likely to be representative of the broader population of *panthi* and bisexual MSM (including those ‘hidden’), and explore their potential importance for the HIV epidemic.

## 2 Methods

### 2.1 Model structure

**Fig. 1:**
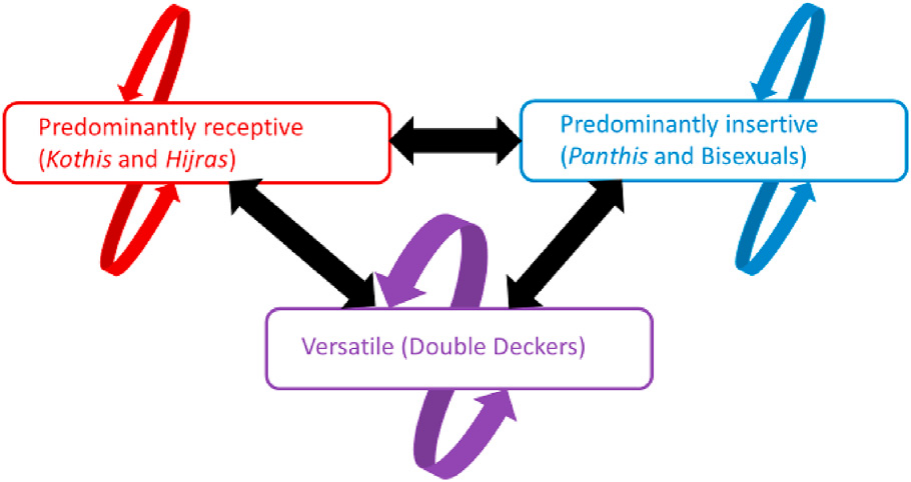
Conceptual representation of model structure: MSM/TGW sexual mixing and HIV transmission (mostly between groups but allowing some within-group mixing as per the data).

A deterministic dynamic compartmental model of HIV and STI transmission was developed (details provided in Appendix A). In brief, the MSM/TGW were grouped into three subgroups according to their sexual identity and predominant role behaviour (Fig. 1): *kothi* and *hijra* (mainly receptive); double deckers (versatile); and *panthis* and bisexuals (mainly insertive).

HIV infection was divided into four stages, with increased infectivity for the first and last stages. MSM/TGW were assumed to cease sexual activity following the last stage of HIV when they developed AIDS (Wawer et al., 2005). MSM/TGW also leave the model as they cease MSM/TGW sexual activity at rate 1/(duration sexually active as MSM/TGW).

Antiretroviral therapy (ART) was modelled as a separate compartment from when it was initially distributed in 2006 (Beattie et al., 2012). Owing to a lack of data on ART coverage for MSM/TGW in Bangalore, we assumed a recruitment rate similar to that used by others (Degenhardt et al., 2010), with 10–50% of eligible MSM/TGW per year being recruited onto ART. Although general population recruitment rates from 2006 to 2009 were lower (3–20%), we expected MSM/TGW to be recruited at a higher rate since a large proportion of the visible MSM/TGW were already linked into HIV care services. Data from Bangalore from that time and soon after also supports this range of ART coverage among MSM/TGW (Mehta et al., 2015; Pickles et al., 2013), although there has been scale up of ART coverage across India in more recent years (National AIDS Control Organisation, 2017a; Pickles et al., 2013). ART was assumed to reduce HIV transmissibility (Cohen et al., 2011) and increase survival (Wandel et al., 2008) in the model.

Two other STIs (HSV-2 and syphilis), and their interaction with HIV, were incorporated into the model. HSV-2 transmission was modelled dynamically in parallel to HIV, assuming a susceptible-infected structure for this lifelong infection (Becker, 2002). HSV-2 reached endemic equilibrium before HIV was seeded. Syphilis prevalence was assumed to remain constant over time. MSM/TGW infected with either syphilis, HSV-2 or both had an increased probability of acquiring or transmitting HIV (Freeman et al., 2006).

As in our prior modelling analysis (Mitchell, Foss, Prudden, et al., 2014), the size of the insertive MSM subgroup was set to balance the total number of insertive and receptive sex acts in the whole MSM/TGW population. The number of receptive sex acts reported by each subgroup were distributed among the three subgroups (as their partners) in proportion to the number of insertive acts reported by each (partner) subgroup. Additional information about how mixing between subgroups was modelled can be found in Appendix B.

The MSM/TGW population size was assumed to remain constant over time, with those MSM/TGW leaving the model being replaced by new susceptible individuals of the same subgroup. The robustness of the model predictions to population growth was explored in the sensitivity analysis (see subsection 2.4.1).

### 2.2 Data for parameterising and fitting the model

HIV, HSV-2 and syphilis prevalence data were obtained from two IBBAs conducted in urban Bangalore (Bill & Melinda Gates Foundation, 2008). The first survey was conducted in 2006 (n = 307) (Brahmam et al., 2008; Saidel et al., 2008) and the second in 2009 (n = 403) (Karnataka Health Promotion Trust, 2013). Data for behavioural parameters were obtained from the IBBA face-to-face interview questionnaires (2006 and 2009) and a special behavioural survey (SBS) conducted among MSM/TGW in Bangalore in 2006 (Phillips et al., 2008). Table 1 provides the behavioural parameters, and their sources and derivation, with further details in Appendix C. Biological parameter values were obtained from the scientific literature and are given in Table 2.

**Table 1:** Behavioural parameter ranges for all three modelling approaches.

Behavioural parameter estimates	Parameter range		References/additional details
	Approach 1	Approaches 2 and 3	
**Baseline consistency of condom use prior to start of data collection (prior to 1998)**	0–25%		Unpublished MSM/TGW data from urban Bangalore using technique from (Lowndes et al., 2010)
**Consistency of condom use during anal sex (2006–2009)**	76.6–98.7%		Data on condom use with “known” and “unknown” MSM/TGW partners. Lower bound from IBBA round 1 2006 (Brahmam et al., 2008; Saidel et al., 2008) and upper bound from IBBA round 2 2009 (Brahmam et al., 2008).
**Upward gradient estimate for increase in condom use until 2006**	6.67–8.57%		IBBA round 1 and round 2 (2006 and 2009) data (Brahmam et al., 2008; Karnataka Health Promotion; Saidel et al., 2008; Karnataka Health Promotion Trust, 2013) used with technique from (Lowndes et al., 2010)
**Percentage of sex acts that are insertive in the versatile (double decker) population**	29–46%		Special Behavioural Survey (SBS) 2006 data (Phillips et al., 2008)
**Percentage of all sex acts that are insertive in the receptive (*kothi* and *hijra*) population**	3–17%		SBS 2006 data (Phillips et al., 2008)
**Percentage of all sex acts that are insertive in the insertive (*panthi* and bisexual) population**	70–88%	50–100%	For Approach 1 used SBS 2006 (Phillips et al., 2008). For Approaches 2 and 3 allowed wide uncertainty bound, but assumed >50% of sex acts are insertive to ensure number of sex acts with receptive and versatile MSM/TGW balance.
**Length of time versatile MSM remain sexually active as MSM**	9.3–18.6 years		Calculated ‘average age of subgroup’ and subtracted ‘mean age of first anal sex’, to give current time as MSM. Upper bound is double current mean time as MSM (Anderson, 2009)
**Length of time receptive MSM/TGW remain sexually active as MSM/TGW**	11.4–22.8 years		Same technique as detailed for versatile MSM (Anderson, 2009)
**Length of time insertive MSM remain sexually active as MSM**	11.8–23.6 years		Same technique as detailed for versatile MSM (Anderson, 2009)
**Estimated population size of versatile MSM group**	2490–15565		IBBA rounds 1 and 2, SBS survey (AE et al., 2008; Brahmam et al., 2008; Karnataka Health Promotion; Saidel et al., 2008; Karnataka Health Promotion Trust, 2013; Phillips). Used subgroup sizes from these surveys to estimate the ratio of double decker to *kothi* and *hijra* population, which ranges 0.4–2.5. Used these values multiplied by 6230 as total number of *kothi* and *hijra* (see next row).
**Estimated population size of receptive MSM/TGW group**	6230–12460		Population size estimation survey provided by Sangama NGO in Bangalore (Bill & Melinda Gates Foundation, 2008; Lowndes et al., 2012). Assumed population size may be double as upper bound.
**Percentage of versatile MSM infected with HIV at start of epidemic**	0–4%		Seed MSM within same range as HIV prevalence among female sex workers (FSWs) in early data from India (Arora, Cyriac, & Jha, 2004)
**Percentage of receptive MSM/TGW infected with HIV at start of epidemic**	0–4%		Seed MSM/TGW within same range as FSWs (Arora et al., 2004)
**Percentage of insertive infected with HIV at start of epidemic**	0–4%		Seed MSM within same range as FSWs (Arora et al., 2004)
**Number of sex acts per year, versatile MSM**	104–312		IBBA round 1 data used, adding sex acts with known and unknown partners (Cáceres, Konda, Pecheny, Chatterjee, & Lyerla, 2006)
**Number of sex acts per year, receptive MSM/TGW**	156–442		IBBA round 1 data used, adding sex acts with known and unknown partners (Cáceres et al., 2006)
**Number of sex acts per year, insertive MSM**	2–208	2–442	Approach 1 used IBBA round 1 data, adding sex acts with known and unknown partners. Lower bound estimate assumed 1 sex act with a man/TGW every six months (as our definition of a sexually active MSM/TGW) (Cáceres et al., 2006). Approach 2 and 3 assumed same lower bound as Approach 1, but increased upper bound to match receptive MSM/TGW to allow for uncertainty.

**Table 2:** Biological parameter ranges for all three modelling approaches, and epidemiological data used for model fitting.

Biological parameter estimates	Parameter range		References
**Start year of HIV epidemic**	1981–1990		The start date for the HIV epidemic in the MSM/TGW population was based on the first identified case in female sex workers (FSWs) in Tamil Nadu in 1986 (Simoes, Babu, Jeyakumari, & John, 1993), but allows for the fact that the epidemic may have started earlier (Gottlieb et al., 1981) (first reported case in the USA) or later (Bollinger, Tripathy, Quinn, & Thomas, 1995) in Bangalore (high rates of HIV reported in STI clinic patients in 1993/1994 including MSM/TGW).
**HIV transmission probability per-anal-sex-act for insertive to receptive MSM/TGW partners**	0.002–0.014		Estimates of the probability of HIV transmission from an insertive to receptive partner (0.008 per sex act, 95% CI 0.002–0.014) were generated from a systematic review (Boily et al., 2009).
**HSV-2 transmission probability per-anal-sex-act for insertive to receptive MSM/TGW partners**	0.00018–0.00074		Brown et al. (2006)
**Efficacy of condoms in protecting against HIV**	74–94%		(Weller & Davis, 2003) multiplied lower bound by IBBA round 2 data on “condom breakage at last sex act” (7.8%) to obtain low efficiency estimate
**Efficacy of condoms in protecting against herpes simplex virus type 2 (HSV-2)**	6–60%		Martin et al. (2009)
**Increase in the infectiousness of HIV during the initial high viraemia stage of HIV**	4.5–18.8		Boily et al. (2009)
**Increase in the infectiousness of HIV during the final high viraemia stage of HIV prior to AIDS**	4.5–11.9		Boily et al. (2009)
**Length of time an individual remains in the initial high viraemia stage of HIV**	1.23–2.9 months		The high-viraemia period following initial HIV infection was estimated from the literature to last for 1.23–2.9 months (Hollingsworth, Anderson, & Fraser, 2008)
**Length of time an individual remains in the long asymptomatic low viraemia stage of HIV (not eligible for ART at that time as CD4** > **200)**	7.35 years		Using (Wandel et al., 2008) which suggests from diagnosis to ART eligibility takes 7.6 years, then assumed this phase of HIV must last 7.6–0.25 years = 7.35 years since the initial high-viraemia period is 0.25 years
**Length of time an individual remains in the short asymptomatic low viraemia stage of HIV (eligible for ART as CD4** < **200)**	3–13 months		Total time in asymptomatic phase is between 7.6 and 8.36 years (Wandel et al., 2008). Therefore, time in shorter asymptomatic phase is between 7.6-7.35 (=0.25 years) and 8.36-7.35 (=1.1 years).
**Length of time an individual remains in the high viraemia stage of HIV prior to AIDS (eligible for ART as CD4** < **200)**	4–14 months		Used (Hollingsworth et al., 2008) in (Wawer et al., 2005) to estimate the number new HIV infections by sexual transmission in AIDS phase so MSM/TGW leave model after this stage.
**Length of time an individual remains sexually active whilst on ART treatment before progressing to AIDS**	0.8–8.4 years		Adjusting for imperfect adherence (Mehta et al., 2015; Wandel et al., 2008)
**Multiplicative reduction in the transmission probability of HIV to insertive versus receptive partner**	0.003–0.5		Used (Jin et al., 2010) as upper bound estimate. Used (Pinkerton, Abramson, Kalichman, Catz, & Johnson-Masotti, 2000) for lower bound.
**Multiplicative reduction in the transmission probability of HSV-2 to insertive versus receptive partner**	0.4–0.67		Brown et al. (2006)
**Multiplicative increase in the probability of acquiring HIV if either partner is infected with an STI (HSV-2 or TP)**	1.2–5.3		Used 1.2 as lower bound reported in systematic review among MSM/TGW (Freeman et al., 2006). For upper bound, used 5.3 (mean) from systematic review (Boily et al., 2009).
**Prevalence of syphilis (TP) in sampled double decker population**	6.7%		Used high titre estimate from IBBA round 2 data (2009), mean value (Karnataka Health Promotion Trust, 2013)
**Prevalence of syphilis (TP) in sampled *kothi* and *hijra* population**	5.1%		Used high titre estimate from IBBA round 2 data (2009), mean value (Karnataka Health Promotion Trust, 2013)
**Prevalence of syphilis (TP) in sampled *panthi* and bisexual population**	2.1%		Used high titre estimate from IBBA round 2 data (2009), mean value (Karnataka Health Promotion Trust, 2013)
**Prevalence of HSV-2 in sampled double decker population**	26.7% (17.2–36.3%)		Mean values and 95% CI range from IBBA round 1 (2006) (Brahmam et al., 2008; Saidel et al., 2008)
**Prevalence of HSV-2 in sampled *kothi* and *hijra* population**	38.7% (31.1–46.2%)		Mean values and 95% CI range from IBBA round 1 (2006), using weighted averages in cases where subgroups were combined (Brahmam et al., 2008; Saidel et al., 2008)
**Prevalence of HSV-2 in sampled *panthi* and bisexual population**	29.6% (18.7–40.5%)		Mean values and 95% CI range from IBBA round 1 (2006), using weighted averages in cases where subgroups were combined (Brahmam et al., 2008; Saidel et al., 2008)
**Prevalence of HIV in sampled double decker population**	12.8% (5.6–20.0%)	12.1% (6.8–17.4%)	Mean values and 95% CI range from IBBA round 1 (2006) in first column (Brahmam et al., 2008; Saidel et al., 2008) and IBBA round 2 (2009) in second column (Karnataka Health Promotion Trust, 2013)
**Prevalence of HIV in sampled *kothi* and *hijra* population**	22.7% (16.2–29.2%)	22.5% (16.2–28.8%)	Mean values and 95% CI range from IBBA round 1 (2006) in first column (Brahmam et al., 2008; Saidel et al., 2008) and IBBA round 2 (2009) in second column (Karnataka Health Promotion Trust, 2013), using weighted averages in cases where subgroups were combined
**Prevalence of HIV in sampled *panthi* and bisexual population**	12.7% (4.7–20.6%)	13.1% (4.4–21.8%)	Mean values and 95% CI range from IBBA round 1 (2006) in first column (Brahmam et al., 2008; Saidel et al., 2008) and IBBA round 2 (2009) in second column (Karnataka Health Promotion Trust, 2013), using weighted averages in cases where subgroups were combined

Estimates of the total MSM/TGW population size for urban Bangalore were based on anonymous polling booth interview data from Karnataka (on “ever” having anal sex with another man), in which participants indicted their response by placing tokens in containers which were not traceable to individuals (Lowndes et al., 2012). The highest estimate (6.6%), from Mysore (the city closest geographically to Bangalore), was used to calculate an upper bound estimate for the total number of men ‘currently’ engaging in sex with men (Lowndes et al., 2012). No lower bound constraint was assumed; this was simply set to zero. This large range created through data extrapolation from ‘ever’ to ‘currently’ MSM also then allowed for the additional subpopulation of TGW.

Increases in condom use over time were modelled using retrospectively estimated condom use trends for the years 1998–2006 detailed in Appendix D (Lowndes et al., 2010).

The model was fit to IBBA data on HIV prevalence from 2006 to 2009, and HSV-2 prevalence from 2006 (detailed in Table 2) (Brahmam et al., 2008; Karnataka Health Promotion; Saidel et al., 2008; Karnataka Health Promotion Trust, 2013).

### 2.3 Three approaches to model fitting and parametric uncertainty analysis

Given the uncertainty ranges on the model parameter estimates (Table 1, Table 2), Latin Hypercube Sampling was used to generate five million combinations of parameter input sets, using uniform distributions for all parameters.

The reports of the number of partners and role preferences among MSM/TGW sampled in the survey suggest that there were likely to be a greater proportion of MSM/TGW who predominantly took the insertive role in anal sex than suggested by the survey (to ‘balance’ the high demand for insertive sex from the typically receptive MSM/TGW in the survey sample). We investigated whether a more rigorous insight into the characteristics of these ‘hidden’ MSM could be gained through comparing three different ‘approaches’, i.e. hypotheses that motivate different prior assumptions or posterior requirements around parameter values.

**Approach 1** assumed that all behavioural and epidemiological data from the IBBAs and SBS for the insertive subgroup were representative of the insertive MSM in the wider population (including the ‘hidden’ MSM), and so the model was both parameterised and fit to the available data from the surveys. For this approach, model runs were classified as model fits if the HIV and HSV-2 prevalence in all three MSM/TGW subgroups were within the 95% confidence intervals (CIs) for the available IBBA data (Table 2).

**Approach 2** assumed that the insertive MSM in the survey were not representative of the ‘hidden’ insertive MSM in the population overall. To explore the uncertainty in the sexual behaviour of the insertive MSM, the relevant behavioural parameter ranges were expanded, namely the total number of sex acts among insertive MSM and the percentage of their sex acts that were insertive (see Table 1 for details). In addition, no fitting constraints were applied to the insertive MSM subgroup, meaning that model fits were determined as parameter sets which gave HIV and HSV-2 prevalence estimates within the 95% CI of the IBBA data for receptive and versatile MSM/TGW only.

**Approach 3** used the same model fits as Approach 2, but added an extra criterion to reject fits if the insertive subgroup had a greater number of sex acts than the receptive group, based on suggestions from previous studies that *Hijra* and *Kothis* have a higher frequency of sex (Brahmam et al., 2008; Phillips et al., 2008).

In all three approaches, no fitting restriction was applied to the proportion of MSM/TGW that were in the insertive subgroup. Instead, the proportion who were insertive was simply determined by the proportions in the other two subgroups and the cap on the total number of MSM/TGW across all three subgroups based on available data as explained in Section 2.2.

### 2.4 Model analyses

The model fits from each approach were used to estimate the HIV prevalence and incidence among all MSM/TGW subgroups, and their population sizes. The contribution of insertive MSM to the broader epidemic among men was estimated by calculating the ratio of the number of HIV-positive insertive MSM to the number of HIV-positive urban males. The number of HIV-positive urban males is simply the product of the population size of urban males and the HIV prevalence in Karnataka for males aged 15–54, estimated by the National Family Health Survey (NFHS) data to be 0.82% in 2006 (National Family Health Survey, 2007).

#### 2.4.1 Sensitivity analysis

Uncertainty in the rate at which eligible MSM/TGW were recruited onto ART may be a limitation to our study, so we conducted a sensitivity analysis in which this term was set to zero (i.e. no ART) to assess the extent to which the model results were affected by the ART assumptions.

In addition, to explore the robustness of the key findings to changes in model structural assumptions, an independent ‘alternative’ model was built by a second modeller and compared to the ‘baseline’ model used by the first modeller. The alternative model did not include STI transmission (as was included in the baseline model), but did incorporate the potential protective effect of male circumcision and population growth (aspects that were not in the baseline model). Further technical details about both models are contained in the Appendices.

## 3 Results

Of the five million model runs, 283 model runs fit within the 95% CI of the HIV/HSV-2 prevalence data among all MSM/TGW subgroups for Approach 1. For Approaches 2 and 3, 470 and 445 model runs, respectively, fit within the 95% CIs of the data for the receptive and versatile MSM/TGW subgroups.

### 3.1 Estimating the HIV prevalence of the ‘hidden’ MSM

For all three approaches, over 98% of model fits suggested the HIV prevalence for the insertive subgroup in 2009 to be below the IBBA 2009 survey-estimated mean HIV prevalence for insertive MSM. This implies that HIV prevalence among insertive MSM in urban Bangalore is lower than among the insertive MSM captured in the survey. Therefore, HIV prevalence among the more ‘hidden’ MSM is likely to be considerably lower than the prevalence among the insertive MSM in the IBBA sample.

**Fig. 2:**
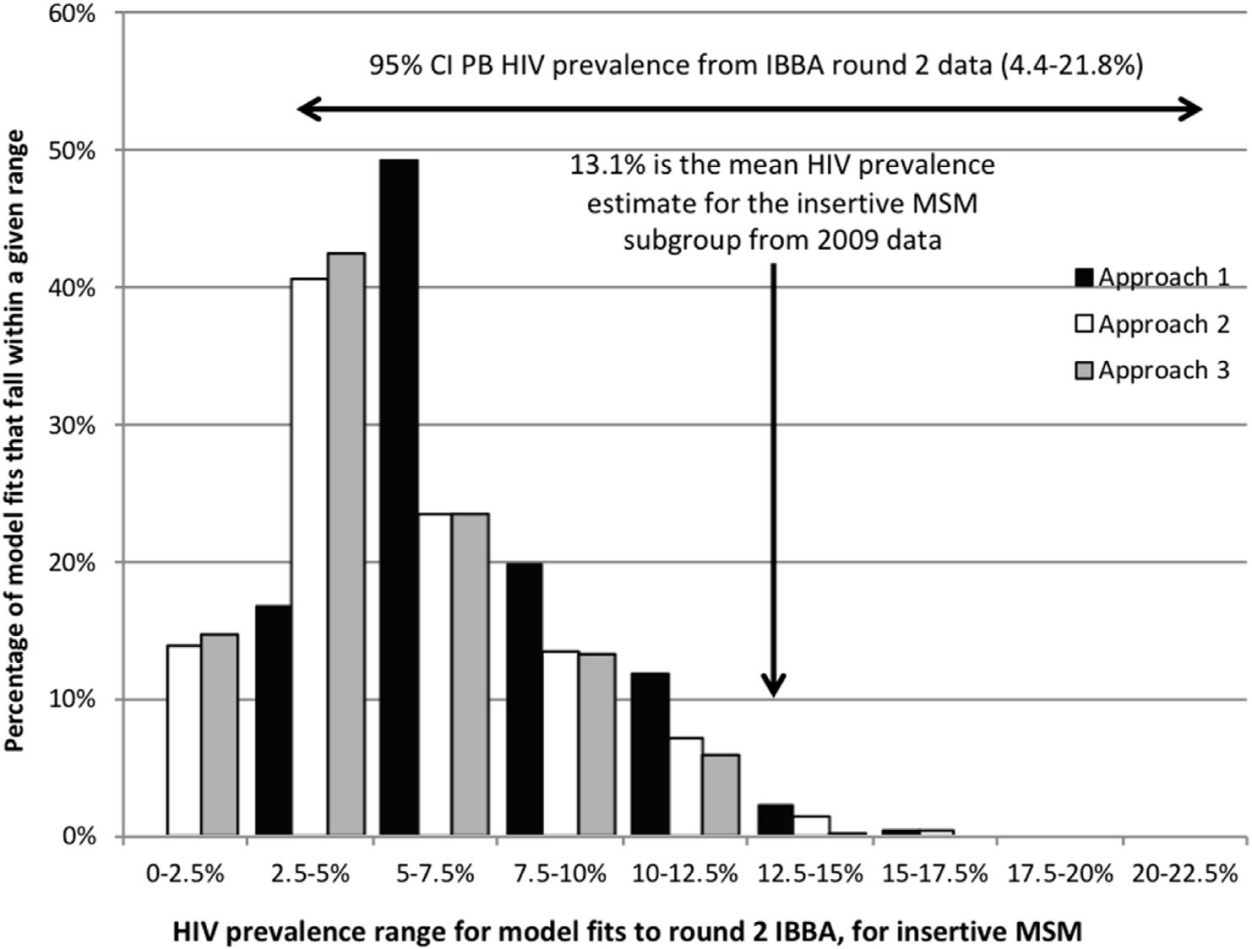
Model-projected HIV prevalence among insertive MSM in 2009 compared with round 2 (2009) IBBA HIV prevalence data for the insertive MSM subgroup, i.e. panthis and bisexuals (PB).

**Fig. 3:**
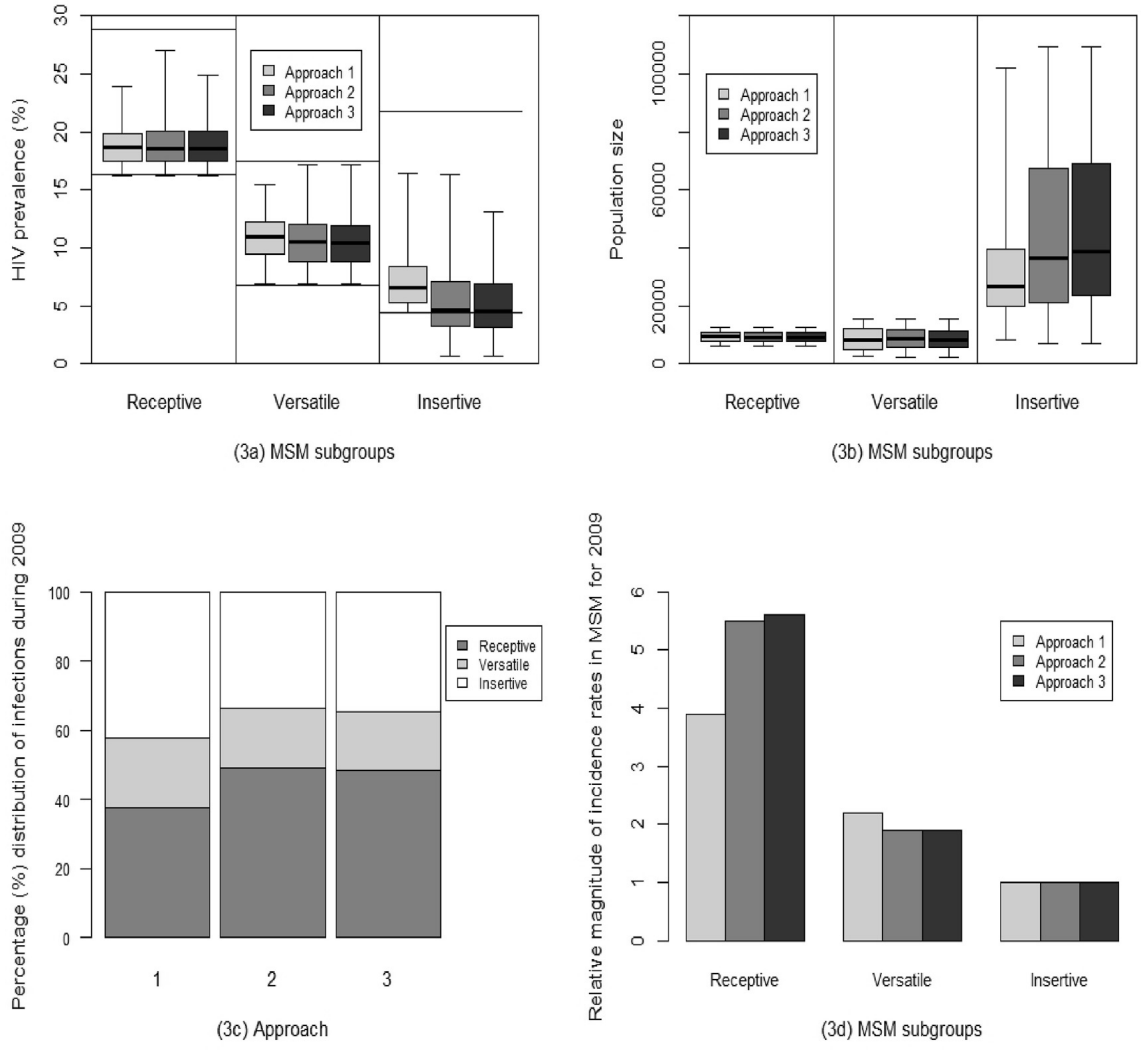
Comparing HIV prevalence, population sizes, and relative prevalence and incidence of infection in each MSM/TGW subgroup. (3a) Comparison of HIV prevalence estimates (median, IQR and range) projected by the model, for the three MSM/TGW subgroups (in 2009) using the three different approaches. The long horizontal lines represent the 95% CI for the IBBA round 2 data for MSM/TGW subgroups. (3b) Model projected estimates of the MSM/TGW subgroup population sizes (median, IQR and range) for the three different approaches. (3c) Model projected percentage distribution of MSM/TGW prevalent HIV infections for the three MSM/TGW subgroups (2009) across the three different approaches. (3d) Comparison of relative magnitude of incidence rates (during year 2009) in the MSM/TGW subgroups (relative to the insertive group), i.e. the number of infections in the versatile and receptive groups for every one infection in the insertive group, across the three different approaches.

Fig. 2 compares the HIV prevalence projections from the model fits for each approach with the IBBA data from the same time point (2009). Approach 1 estimates a median HIV prevalence among the insertive MSM of 6.5% (IQR 5.3–8.3%), while Approaches 2 and 3 give lower median estimates of 4.7% (IQR 3.3–7.1%) and 4.5% (IQR 3.1–6.9%), respectively. This is further illustrated in Fig. 3a through the comparison of HIV prevalence estimates (median, IQR and range) projected by the model for all three MSM/TGW subgroups, for the different model fitting approaches.

### 3.2 Estimating the size of the ‘hidden’ and total MSM/TGW populations

Approach 1 estimates a median total MSM/TGW population of 44,700 (IQR 37,700-58,600), with the insertive MSM group accounting for 61% (IQR 52–70%) of the total MSM/TGW population. In comparison, the median estimates were 55,600 (IQR 39,100-83,800) for Approach 2 and 58,600 (IQR 41,800-85,600) for Approach 3, with the insertive MSM subgroup comprising 68% (IQR 55–78%) and 69% (IQR 56–79%) of all MSM/TGW, respectively (Fig. 3b). The flexible Approaches 2 and 3 (which allowed a wider range of uncertainty) suggest that there are moderately more ‘hidden’ insertive MSM than might be inferred from simply parameterising and fitting the model to the survey data on insertive MSM (as was done in Approach 1). Across all three approaches, the model suggests a much larger percentage of MSM/TGW are in the insertive subgroup (61–69%) compared to the IBBA 2009 sample which contained just 15% in this subgroup (Karnataka Health Promotion Trust, 2013).

### 3.3 Comparing the percentage distribution of HIV infections across each MSM/TGW subgroup and the relative magnitude of incidence rates in MSM/TGW subgroups

Modelled prevalence projections for 2009 were used to estimate the overall percentage of prevalent infections occurring among the insertive MSM compared with the receptive and versatile subgroups (Fig. 3c). Approach 1 estimates that 41.4% of prevalent infections were among insertive MSM, the highest among all the MSM/TGW subgroups. However, Approaches 2 and 3, respectively, provide slightly lower estimates of 32.9% and 33.8%, with the highest proportion of infections for these scenarios being amongst the receptive subgroups (49.1% and 48.5%).

The relative magnitude of incidence rates in MSM/TGW subgroups (relative to the insertive group) was also estimated for 2009 through comparing the total annual number of incident infections that year (Fig. 3d). For Approach 1, we project that 3.9 times as many infections occurred in receptive MSM/TGW compared to the insertive group, while the versatile subgroup had 2.2 times more infections. In comparison to the insertive group, Approaches 2 and 3 estimated the receptive MSM/TGW subgroup to have, respectively, 5.5 and 5.6 times more annual infections, while the versatile subgroup had 1.9 times more (by both approaches).

### 3.4 Projecting the contribution of insertive MSM to the broader HIV epidemic in Bangalore

The model was used to estimate the overall prevalence of HIV among MSM/TGW in 2009. The median estimate for Approach 1 was 9.8% (IQR 8.5–11.5%), for Approach 2 it was 8.4% (IQR 6.2–10.5%) and for Approach 3 it was 8.2% (IQR 6.1–10.2%). All three estimates were substantially lower than the overall estimate from the IBBA 2009 data of 17.0% (95% CI 13.4–21.0%). Our modelling results suggest that insertive MSM account for 10–20% of all prevalent HIV infections among urban males aged 15–49.

#### 3.4.1 Sensitivity analysis

Removing ART in our sensitivity analysis had minimal effect on the model findings (increasing the median HIV prevalence by no more than 0.3% for the insertive MSM in all three model approaches).

The key findings, that the insertive MSM subgroup was larger in size but had lower HIV prevalence than suggested by the IBBA sample, were robust across both models, insensitive to assumptions about STIs, circumcision and population growth. In fact, the alternative model projected a larger bias in the empirical estimates, suggesting that the insertive MSM subgroup population size may be even larger and their HIV prevalence even lower than was suggested by the baseline model.

## 4 Discussion

We have shown that our mathematical modelling technique can be used with triangulation of survey data to infer characteristics of more ‘hidden’ MSM and explore their potential importance for the HIV epidemic. Our model projections across all three different fitting approaches suggest that HIV prevalence among insertive MSM (including those ‘hidden’) was less than half of that recorded in the IBBA 2009 survey (4.5–6.5% versus 13.1%), while the size of the insertive subgroup was over four times larger (61–69% of all MSM/TGW versus 15%). The three approaches also consistently indicated that insertive MSM accounted for 10–20% of all prevalent HIV infections among urban males aged 15–49.

### 4.1 Our findings in context

The survey-estimated HIV prevalence among *panthi* and bisexual (insertive) MSM found at ‘hot-spot’ cruising sites was not representative of insertive MSM across Bangalore: our modelling suggests that the survey over-estimated HIV prevalence in this subgroup.

Mapping studies previously conducted in Bangalore for MSM/TGW have tended to only capture receptive and versatile individuals (Karnataka State AIDS Prevention Society, 2012), and no study has attempted to enumerate the total MSM/TGW population size including the mostly ’hidden’ insertive group. Social media data from several other countries suggests that official size estimates of MSM/TGW populations are typically underestimates (Baral et al., 2018). Earlier evidence indicated that, in southern Asia, 6–12% of men report ever having had sex with another man in their lifetime and about half as many reported sex with a man in the past year (Cáceres et al., 2006). Using this conversion factor of a half, with polling-booth survey data from other regions in Karnataka estimating that 4–6.6% of males have ever engaged in anal sex with another man (Lowndes et al., 2012), implies that 2–3.3% had sex with a man in the past year: our model estimates closely agree.

Another study, across several different states in India, estimates that about 1 in 10 rural men have had unprotected anal sex with another man in the past year, and that these men also report high numbers of female partners with whom they engage in anal sex as well (Solomon et al., 2015; Verma & Collumbien, 2004). This suggests a high risk of bridging infections between MSM and the heterosexual population (Kumar et al., 2014; Ramakrishnan et al., 2015; Solomon et al., 2015; Verma & Collumbien, 2004). That said, another study concluded that if MSM/TGW were exposed to HIV intervention programmes, in-particular condom distribution and condom demonstrations, then this resulted in safer sexual practices (Mitchell, Foss, Ramesh, et al., 2014). Similarly, a pre-planned, causal-pathway-based modelling analysis indicated that behavioural interventions for female sex workers and MSM/TGW across 24 southern Indian districts averted substantial numbers of HIV infections (Pickles et al., 2013).

### 4.2 Strengths and limitations

The main limitation to our study is that much of the data and analysis focuses on 2009, meaning that the findings are now less relevant to that local context. Over the past decade there has been increased tolerance and acceptance in India, especially in large cities, although legal reforms have oscillated from decriminalising homosexuality in 2009, making it illegal again in 2013, then legalising in 2018 (Beyrer et al., 2016; Harris, 2013; Subramanian, 2018; Timmons & Kumar, 2009). There has also been much more extensive use of social media to connect for sex (Das et al., 2019).

However, the insights gained here could have broader methodological implications. We have demonstrated a technique in which survey data from a more ‘visible’ population was used with mathematical modelling and triangulation techniques (for ‘balancing’ reports of the number of partners and role preferences of different MSM/TGW subgroups), to infer characteristics of a more ‘hidden’ or less sampled MSM/TGW subgroup.

Modelling techniques such as these could be used in other situations, when there may be a large degree of uncertainty in some sections of a network of disease spread, for example due to missing data or concerns about the representativeness of the data. These techniques add further to the work of others in using imperfect data from multiple sources to infer unknowns indirectly by applying Bayesian evidence synthesis methods (Birrell et al., 2011; Rosinska, Gwiazda, De Angelis, & Presanis, 2016).

The analysis is also somewhat limited by the lack of data about sexual mixing patterns, although previous modelling has shown that, for this setting, HIV prevalence projections are largely unaffected by the underlying mixing assumptions (Mitchell, Foss, Prudden, et al., 2014). Insufficient data on other factors or inconsistent data due to different data collection methods (Phillips et al., 2013) also presented a challenge, leading us to make necessary simplifying assumptions in the model to match data availability (Garnett & Anderson, 1996) or having to obtain some model parameter estimates from nearby districts or state-level data. However, the sensitivity analyses showed that removal of ART or STIs, or incorporation of male circumcision or population growth, made little difference to our main findings.

### 4.3 Recommendations and remaining questions

The findings illustrate the need for further and updated research into the population of ‘hidden’ MSM. An improved understanding of the population size and sexual behaviour of all MSM/TGW is vitally important for implementing effective HIV interventions to reduce HIV risk among MSM/TGW and onward transmission to their female partners. In fact, a recent paper concluded that underestimations of the size of the MSM population likely limited the impact of their integrated HIV services across 27 Indian sites (Solomon et al., 2019). With most intervention programmes focusing on ‘visible’ MSM/TGW, the ‘hidden’ MSM could be considered as a potentially marginalised group that should be carefully considered rather than overlooked.

Could early HIV treatment as prevention or pre-exposure prophylaxis among the ‘visible’ MSM/TGW be a cost-effective strategy to reduce the risk of transmission to ‘hidden’ MSM and onward spread of infection to their female partners and the general population, especially since the ‘visible’ MSM/TGW are more easily reached, at higher risk of acquiring HIV as predominantly receptive, and may be more sexually active (Mitchell et al., 2016)? Alternatively, might such a strategy have limited impact on the broader epidemic if the large population of ‘hidden’ MSM are not reached directly by these initiatives? It is possible that strategies to reach the ‘hidden’ MSM may also inadvertently reach lower-risk men since the ‘hidden’ MSM may not be reachable by MSM/TGW programming, so reducing the efficiency of these strategies (Mitchell et al., 2016).

Others have successfully recruited more *panthis* (45% of MSM/TGW) through respondent-driven sampling and have advocated to use such methods to increase the representation of these ‘hidden’ MSM in survey data and intervention reach (Solomon et al., 2015), although there remain challenges with the implementation of either respondent-driven sampling or time-location sampling and both are still seen as the most appropriate for such populations (MacCarthy et al., 2016). However, a major challenge has been the high levels of stigma and discrimination experienced by individuals engaging in same-sex relationships, limiting their contact with services (Chakrapani, Newman, Shunmugam, McLuckie, & Melwin, 2007). Offering some hope, a recent study suggests that peer mobilisation via social media can reach more ‘hidden’ MSM for HIV services (Das et al., 2019).

## 4.4 Conclusions

Our analysis shows that mathematical modelling can be combined with data from ‘visible’ MSM/TGW to provide insights into the characteristics of under-sampled ‘hidden’ MSM, thus providing a better characterisation of the overall MSM/TGW population. A greater understanding of the sexual behaviour of all MSM/TGW is important for effective HIV programming and for estimating the impact that these programmes may have. There are also important broader public health implications from this work: a hidden subgroup with a lower infectious disease prevalence than more visible subgroups, has the potential to contain more infections, if the hidden subgroup is considerably larger in size.

## Declaration of competing interest

The authors declare that they have no conflict of interests.

## Figures and Tables

**Fig. A1:**
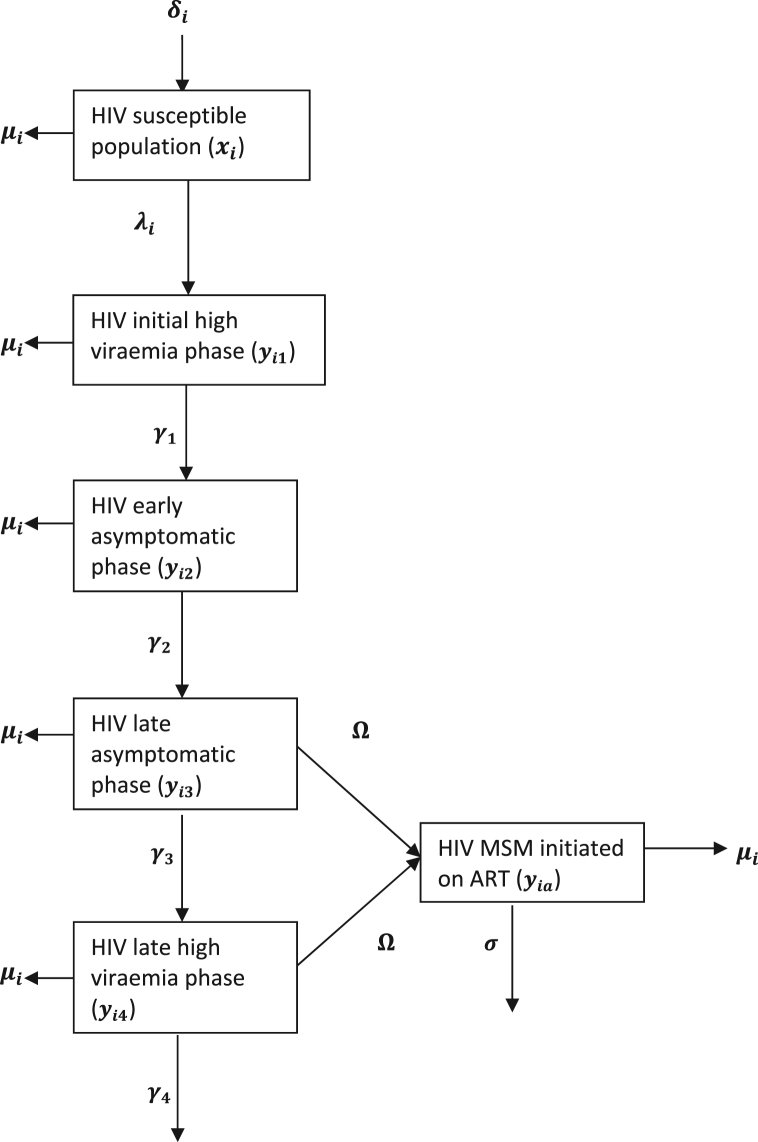
Schematic representation of model.

**Fig. D1:**
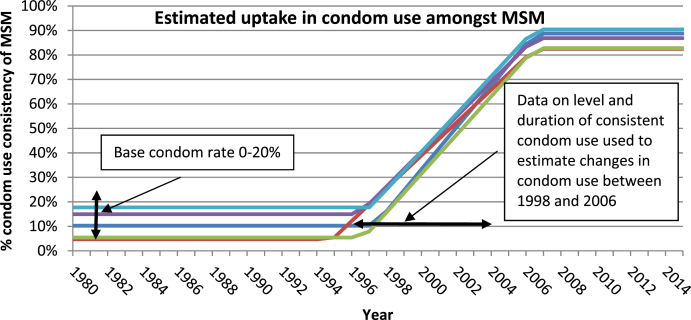
Estimated trends in condom use among MSM.

## Appendix A Model description

As shown in Fig. A1, uninfected MSM ($x_{i}$) become infected with HIV at a per capita rate ($\lambda_{i}$) and move into a short initial high viraemia stage of HIV ($y_{i1}$). Here i=0,1or2 to denote the 3 different MSM/TGW subgroups modelled; those who are predominantly receptive (i=0), those who are predominantly insertive (i=1), and those who take either role (i=2). After the initial high viraemia stage, MSM/TGW progress at a rate $\gamma_{1}$ into a longer asymptomatic stage ($y_{i2}$) in which their probability of transmitting HIV is lower. From here they advance at a rate $\gamma_{2}$ to a late asymptomatic phase of HIV, $y_{i3}$, at which point they are eligible for ART treatment (CD4<200). If they are not initiated on ART they advance, at rate $\gamma_{3}$, to the late stage of high viraemia HIV infection, prior to development of AIDS ($y_{i4}$). At this late stage they are also eligible for ART treatment. The remaining MSM/TGW in this fourth phase of HIV progress to AIDS at a rate $\gamma_{4}$ and leave the model, assuming at the same time they also cease sexual activity (Wawer et al., 2005).

Initiation of ART treatment began in Bangalore in 2006, therefore MSM/TGW become eligible for treatment only from this point onwards in the model. In order to estimate the uptake of ARTs, a method similar to that used by others (Degenhardt et al., 2010) was employed so that 10–50% of eligible MSM/TGW population in the specified compartments (late asymptomatic or late high viraemia phases) were initiated on ART per year (Ω). Within each MSM/TGW subgroup i, MSM/TGW that initiate ART treatment are assimilated into one treatment compartment ($y_{ia}$), with the effect of treatment both extending their life-expectancy (Wandel et al., 2008) and reducing their likelihood of HIV transmission (Cohen et al., 2011). This group leave the model at a rate σ, as it is assumed that they cease sexual activity as they go on to develop AIDS, e.g. through poor adherence to ART (Mehta et al., 2015; Wandel et al., 2008).

MSM/TGW also leave all compartments at a rate $\mu_{i},$ as they cease MSM/TGW sexual activity at rate 1/(duration sexually active as MSM/TGW). In the baseline model, all MSM/TGW leaving the model due to cessation of MSM/TGW sexual activity after a period of time $\left(\mu_{i}\right),$ or through progression to AIDS $\left(\gamma_{4}\;or\;\sigma \right)$, are replaced by $\delta_{i}$ MSM/TGW susceptible to both HIV and HSV-2, to maintain a constant population size for each subgroup. In the alternative model, this replacement is also made but there is an additional constant recruitment rate (proportional to the initial total population size) incorporated into $\delta_{i}$ to sustain a slowly growing total population in line with data (World Population Review, 2019).

STIs are included in the baseline model only (i.e. there are no STIs in the alternative model). In the baseline model, herpes simplex virus type 2 (HSV-2) infection is modelled simply as MSM/TGW being either susceptible or infected, with per capita rate of HSV-2 infection being denoted by $\lambda_{vi}$. Since HSV-2 is a lifelong infection, once MSM/TGW are infected they remain in this compartment until they cease MSM/TGW sexual activity and exit the model. HSV-2 treatment is not explicitly accounted for in the model, however the estimate for the HSV-2 transmission probability in the model is low to reflect the likelihood of MSM/TGW receiving treatment which reduces shedding. In the baseline model, the probability of either partner having one or both of HSV-2 or syphilis (TP) is calculated in order to obtain a single ‘STI prevalence’ value ($s_{ji}$). This $s_{ji}$ is multiplied by a STI cofactor, κ, facilitating HIV transmission (through increased susceptibility and/or infectivity). The cofactor therefore describes how the risk of HIV infection is increased if either partner has an STI.

## Differential equations

(A.1) $$\frac{dx_{i}}{dt}=\;\delta_{i}-\;x_{i}\left(\lambda_{i}+\;\mu_{i}\right)$$

(A.2) $$\frac{dy_{i1}}{dt}=x_{i}\lambda_{i}\;-y_{i1}\left(\gamma_{1}+\mu_{i}\right)$$

(A.3) $$\frac{dy_{i2}}{dt}=\;y_{i1}\gamma_{1}-y_{i2}\left(\gamma_{2}+\;\mu_{i}\right)$$

(A.4) $$\frac{dy_{i3}}{dt}=\;y_{i2}\gamma_{2}-y_{i3}\left(\gamma_{3}+\;\text{Ω}+\mu_{i}\right)$$

(A.5) $$\frac{dy_{i4}}{dt}=\;y_{i3}\gamma_{3}-y_{i4}\left(\gamma_{4}+\;\text{Ω}+\mu_{i}\right)$$

(A.6) $$\frac{dy_{ia}}{dt}=\;y_{i3}\text{Ω}+y_{i4}\text{Ω}-\;y_{ia}\left(\sigma +\;\mu_{i}\right)$$

where (with time-dependencies suppressed to simplify notation):

$x_{i}$ denotes the number of MSM/TGW in subgroup i=0,1or2 (based on their predominant role) who are susceptible to HIV.

$y_{i1}$ denotes the number of MSM/TGW in subgroup i who are in phase 1 (initial high viraemia phase) following initial HIV infection.

$y_{i2}$ denotes the number of MSM/TGW in subgroup i who are in phase 2 (early asymptomatic phase of HIV) with CD4 cell count >200 cells/mm^3^.

$y_{i3}$ denotes the number of MSM/TGW in subgroup i who are in phase 3 (late asymptomatic phase of HIV) with CD4 cell count <200 cells/mm^3^.

$y_{i4}$ denotes the number of MSM/TGW in subgroup i who are in phase 4 (late high viraemia phase) prior to AIDS.

$y_{ia\;}$ denotes the number of MSM/TGW in subgroup i who are on ART.

$\delta_{i}$ denotes the number of men entering the MSM/TGW subgroup i. In the baseline model, this is set to equal the sum of the number of MSM/TGW leaving that subgroup to maintain a constant population size. In the alternative model, this replacement of those leaving is also made but there is an additional constant recruitment rate incorporated into this term as well to maintain a slowly growing total population.

$\mu_{i}$ denotes the rate at which MSM/TGW in subgroup i cease sex with other men for reasons other than HIV-related morbidity or mortality and is calculated as 1/(duration sexually active as MSM/TGW).

$\gamma_{h}$ denotes the rate at which MSM/TGW leave individual HIV stage compartments to progress through the different stages of HIV, where h represents the different phases of HIV (h=1,2,3or4).

Ω denotes the proportion of MSM/TGW with CD4 cell count <200 cells/mm^3^ who are initiated on ART treatment per year. (This term was set to zero as part of the sensitivity analysis to explore the extent to which the model results were affected by the ART assumptions).

*σ* denotes the rate at which MSM/TGW cease sexual activity (as progress to AIDS) when on ART.

$\lambda_{i}\left(t\right)$ denotes the annual per capita force of infection for HIV transmission for MSM/TGW in subgroup i, and is given by the following formula for the baseline model:

(A.7) $$\lambda_{i}=\left\{c_{i}\left(1-p_{i}\right)\left(1-ef\right)\right\}\overset{2}{\underset{j=0}{\sum }}\left[\rho_{ji}B_{ji}\left(\kappa s_{ji}+1-s_{ji}\right)\right]+\left\{c_{i}p_{i}\left(1-ef\right)\right\}\overset{2}{\underset{j=0}{\sum }}\left[\left(1-\rho_{ji}\right)\beta_{ji}\left(\kappa s_{ji}+1-s_{ji}\right)\right]$$

where:

$c_{i}$ denotes the number of sex acts per year for a given MSM/TGW subgroupi.

$p_{i}$ denotes the proportion of sex acts that are insertive for a given MSM/TGW subgroupi.

e denotes the per sex act efficacy of condoms.

f denotes the proportion of sex acts in which condoms are used.

$\rho_{ji}$ denotes the proportion of receptive sex acts MSM/TGW in subgroup i have with the subgroup *j.*

$B_{ji}\left(t\right)$ denotes the HIV transmission probability from an insertive partner multiplied by the HIV prevalence in that partner population (factoring in different multiplicative cofactors for the increased risk of transmission during the high viraemia stages $y_{i1}$ and $y_{i4}$). This equates to the proportion of partnerships with an insertive partner that will result in HIV transmission (where j=0,1or2 represents the three different MSM/TGW subgroups in the partner population of MSM/TGW in subgroup i=0,1or2).

$\beta_{ji}$ denotes the HIV transmission probability from a receptive partner multiplied by the HIV prevalence in that partner population (factoring in different multiplicative cofactors for the increased risk of transmission during the high viraemia stages $y_{i1}$ and $y_{i4}$). This equates to the proportion of partnerships with a receptive partner *j* that will result in HIV transmission.

$s_{ji}=1-\left(1-u_{v}\right)\left(1-u_{t}\right)$ gives the probability that, within a sexual partnership, at least one individual has an STI, where $u_{v}$ is the prevalence of HSV-2 and $u_{t}$ is the prevalence of syphilis. (This term was only included in the baseline model, not the alternative model).

κ denotes the multiplicative increase in the per sex act probability of HIV transmission in the presence of either STI (HSV-2 or syphilis). (This term was only included in the baseline model, not the alternative model).

In the alternative model, $\lambda_{i}\left(t\right)$ is given by:

(A.8) $$\lambda_{i}=\left\{c_{i}\left(1-p_{i}\right)\left(1-ef\right)\right\}\overset{2}{\underset{j=0}{\sum }}\left[\rho_{ji}B_{ji}\right]+\left\{c_{i}p_{i}\left(1-ef\right)\left(1-\psi \omega \right)\right\}\overset{2}{\underset{j=0}{\sum }}\left[\left(1-\rho_{ji}\right)\beta_{ji}\right]$$

where:

ψ denotes the per sex act efficacy of circumcision in protecting the insertive partner, set at 60% (Friedman et al., 2016; Sanchez et al., 2011). (This term was only included in the alternative model, not the baseline model).

ω denotes the proportion of MSM/TGW who are circumcised, which was set to 0.135 based on Indian data (Morris et al., 2016). (This term was only included in the alternative model, not the baseline model).

## Appendix B Mixing matrix equations

The mixing among MSM/TGW was calculated as per the method described in earlier work (Mitchell, Foss, Prudden, et al., 2014). Using this method, the overall numbers of insertive ($Ins_{i}$) and receptive ($Rec_{i}$) acts for each subgroup i were calculated as:

(B.1) $$Ins_{i}=N_{i}c_{i}p_{i}$$

(B.2) $$Rec_{i}=N_{i}c_{i}\left(1-p_{i}\right)$$

where for each identity subgroup (i=0,1or2), $N_{i}$ denotes population size, and the other parameters are defined above.

Balancing was achieved by altering the subgroup size of the predominantly insertive MSM ($N_{1}$) to give an equal number of insertive and receptive acts across the whole population. If the total numbers of insertive and receptive acts in the whole population balance, then:

(B.3) $$Ins_{0}+Ins_{1}+Ins_{2}=Rec_{0}+Rec_{1}+Rec_{2}$$

Substituting (1) and (2) into (3) and re-arranging gives:

(B.4) $$N_{1}=\frac{N_{0}c_{0}\left(1-2p_{0}\right)+N_{2}c_{2}\left(1-2p_{2}\right)}{c_{1}\left(2p_{1}-1\right)}$$

Mixing probabilities between the different groups were calculated using proportionate mixing (Gupta, Anderson, & May 1989). The probability that an individual has a receptive act with an individual in group j, given that they have a receptive act, is calculated as the number of insertive acts offered by group j divided by the number of insertive acts offered by the whole population:

(B.5) $$\;R_{\rho ,j}=\frac{Ins_{j}}{Ins_{0}+Ins_{1}+Ins_{2}}$$

The insertive mixing probability is calculated in a similar way:

(B.6) $$I_{\rho ,y}=\frac{Rec_{j}}{Rec_{0}+Rec_{1}+Rec_{2}}$$

## Appendix C Additional calculations for the number of sex acts and the proportion of these that are insertive

To estimate the number of sex acts per year, data from the IBBA round 2 (2009) were used, based on responses to questions on the number of sex acts with known and unknown male sexual partners in the past week summed together and multiplied by the number of weeks per year. Because the data were highly skewed, the median and inter-quartile ranges were used rather than the mean estimate and 95% CIs. In this instance the lower bound for the predominantly insertive population was fixed at two (i.e. one sex act with a man/TGW in a six-month period), assuming that MSM/TGW having sex with other men less frequently than this have negligible effect on the HIV transmission dynamics among MSM/TGW overall.

The proportion of all sex acts which were insertive for each MSM/TGW subgroup was estimated using data from the Bangalore SBS. Data on the number of MSM/TGW within a subgroup who have sex with a certain partner type (commercial/non-commercial, known or unknown, providing the sample size of MSM/TGW was greater than ten) was used along with the number of insertive sex acts, out of the last ten, that were reported with those partner types.

## Appendix D Condom reconstruction

Survey data from 2006 to 2009 (IBBA rounds 1 and 2), on the percentage of MSM/TGW (all subgroups) reporting condom use in their last sex act was sampled to estimate upper and lower bounds to gain an estimate for condom use in 2006 which was then applied to all years thereafter. Since condom use was reported to be higher in 2009 we used the upper 95% CI bound as an upper estimate and the lower 95% CI from the 2006 survey data as a low estimate. Changes in condom use between 1998 and 2006 were estimated using reconstructed condom use slopes based on previous analysis (Lowndes et al., 2010). Specifically, using the estimated slope, condom use was projected backwards in time until it reached the base condom rate, which was sampled from a range between 0% and the value calculated for 1998 using the condom reconstruction (Fig. D1).
